# Can live weight be used as a proxy for enteric methane emissions from pasture-fed sheep?

**DOI:** 10.1038/srep17915

**Published:** 2015-12-09

**Authors:** J. M. Moorby, H. R. Fleming, V. J. Theobald, M. D. Fraser

**Affiliations:** 1 Institute of Biological, Environmental and Rural Sciences, Gogerddan, Aberystwyth SY23 3EE, UK

## Abstract

To test the hypothesis that sheep live weight (LW) could be used to improve enteric methane (CH_4_) emission calculations, mature ewes of 4 different breeds representative of the UK sheep industry were studied: Welsh Mountain, Scottish Blackface, Welsh Mule and Texel (n = 8 per breed). The ewes were housed and offered *ad libitum* access to fresh cut pasture of three different types, varying in digestibility: (a) a relatively high digestibility monoculture of perennial ryegrass (*Lolium perenne*), (b) a medium digestibility permanent pasture comprising a range of grass species, and (c) a relatively low digestibility native grassland pasture comprising mainly *Molinia caerulea*. Individual LW, feed dry matter intake (DMI), and CH_4_ emissions in chambers were measured. The linear functional relationship between DMI and CH_4_ emissions was positive (*r* = 0.77) with little breed effect. The relationships between LW and DMI, and LW and CH_4_ emissions were also positive but weaker, regardless of pasture type. It is concluded that change to LW was a poor indicator of DMI and has limited value in the prediction of enteric CH_4_ emissions from mature ewes.

The agricultural livestock industry is tasked with reducing its contribution to global environmental problems^1^. In terms of greenhouse gas (GHG) emissions, it accounts for 37% of anthropogenic methane (CH_4_), with most of this arising from enteric fermentation by ruminants. At the same time rising demand for meat and dairy products means livestock numbers are increasing across the globe^2^. In 2013 the global sheep population was 1,162 million head, an increase of 4% since 2009^3^, and sheep numbers have been forecast to increase 60% by 2050^2^. Equivalent global estimates of annual enteric CH_4_ emissions from sheep computed at Tier 1 following the Intergovernmental Panel on Climate Change (IPCC) 2006 Guidelines for National Greenhouse Gas [GHG] Inventories^4^ were 6,305 kilotonnes, or 132,409 kilotonnes CO_2_e^3^. Tier 1 is the most simplified approach to accounting that relies on default emission factors (EFs)^4^, and takes no account of variables such as diet type, feed intake, animal breed and body size. Furthermore, the range of supporting studies with relevant data to develop the 2006 default Tier 1 EFs for sheep was comparatively limited, and many of the observational data that these have been based upon have been collected from breeds or forages unrepresentative of pastures grazed within the UK and Northern Europe^5^,^6^,^7^,^8^. Improved inventories can be achieved by the use of Tier 2 methodologies, which employ locally-derived EFs relevant to country-specific production systems, and allow for greater accuracy and precision in CH_4_ emission estimates. Improving the precision of emission data reporting is essential if key factors influencing the emission rates from alternative livestock production systems are to be identified and the effectiveness of associated mitigation strategies quantified.

Selection following domestication has led to sheep being adapted to thrive in diverse conditions. Smaller, hardier sheep breeds tend to be found in marginal (typically hill or mountain) areas, and it is possible that physiological or behavioural differences^9^,^10^ may result in these animals utilising low-quality native pasture more efficiently than lowland breeds. In contrast, larger, more productive modern (typically lowland) breeds and cross-breeds should theoretically partition relatively more feed nutrients towards productive purposes. It is well known that CH_4_ emissions tend to increase as feed dry matter intake (DMI) increases (e.g.^5^,^11^,^12^,^13^), i.e. the more a ruminant animal eats, the more CH_4_ is produced. Feed intake in sheep is related to some extent to body size^14^,^15^, and therefore larger sheep may be expected to eat more and therefore produce more CH_4_^16^. In this study the relationship between body size and CH_4_ emissions was defined, with mature ewes of differing modern sheep breeds being offered herbage cut from low, medium and high digestibility grass-dominated swards. It tested the hypothesis that body mass and associated allometric relationships determine enteric CH_4_ production at the individual sheep level, regardless of breed type. If such a relationship existed it could be used to refine future EFs for sheep for Tier 2 inventory reporting mechanisms in the UK and other temperate countries with pasture-based sheep production systems.

## Results

### Feeds offered

Thirty-two mature barren (i.e. not pregnant or lactating) ewes of 4 different breeds (Welsh Mountain, Scottish Blackface, Welsh Mule (Welsh Mountain × Border Leicester) and Texel; n = 8 per breed) were each offered 3 contrasting pasture-only diets (a perennial ryegrass monoculture; a grass-species-dominated permanent pasture; and a *Molinia caerulea*-dominated native pasture, hereafter referred to as Molinia). The ryegrass, permanent pasture and Molinia forages fed during the experiment were characterised as having high, medium and low organic matter digestibility (DOMD) in the feed dry matter (DM) respectively, with the Molinia having a substantially higher fibre concentrations that the other two sward types (Table 1). Conversely the ryegrass pasture was characterised by much higher concentrations of water soluble carbohydrates (WSC) than the permanent pasture and Molinia.

### Role of breed type in influencing voluntary intake and *in vivo* digestibility

The live weight (LW) of the ewes ranged from about 40 kg to over 70 kg (Table 2). Intakes were relatively high when offered ryegrass, at over 1 kg DM d^−1^ for all breed types (Table 2). On this forage type a breed effect of DMI remained once the results were expressed on a metabolic LW (MLW) basis, with the intakes recorded for the Welsh Mountain and Scottish Blackface ewes being the highest and lowest respectively. However, apparent DM digestibility was higher for the forage consumed by the Scottish Blackface ewes, and consequently digestible DMI (DDMI) was similar for all breed types when expressed on a MLW basis. Voluntary intakes on the permanent pasture and Molinia were somewhat lower than they had been on the ryegrass (Table 2). A breed effect on DM digestibility was again recorded when the ewes were offered permanent pasture, with once more the highest value being recorded for the Scottish Blackface ewes. However, there was no effect on DMI or DDMI when expressed on a metabolic LW basis. When offered Molinia there was no effect of breed type on DM digestibility, DMI or DDMI when the results were expressed on a MLW basis.

### Role of breed type in influencing CH_4_ emissions

There was no effect of breed type on the quantity of CH_4_ emitted when the ewes were offered ryegrass (Table 3), but a breed effect was seen on the amount of CH_4_ emitted per kilo DMI when offered the permanent pasture. Only on Molinia was there a breed effect on grams of CH_4_ emitted per head per day. When the results were expressed as CH_4_-energy/feed gross energy intake (Y_m_) a breed effect was recorded only when the ewes were offered the permanent pasture.

### Effect of LW on feed intake and CH_4_ emissions

**Figure 1:**
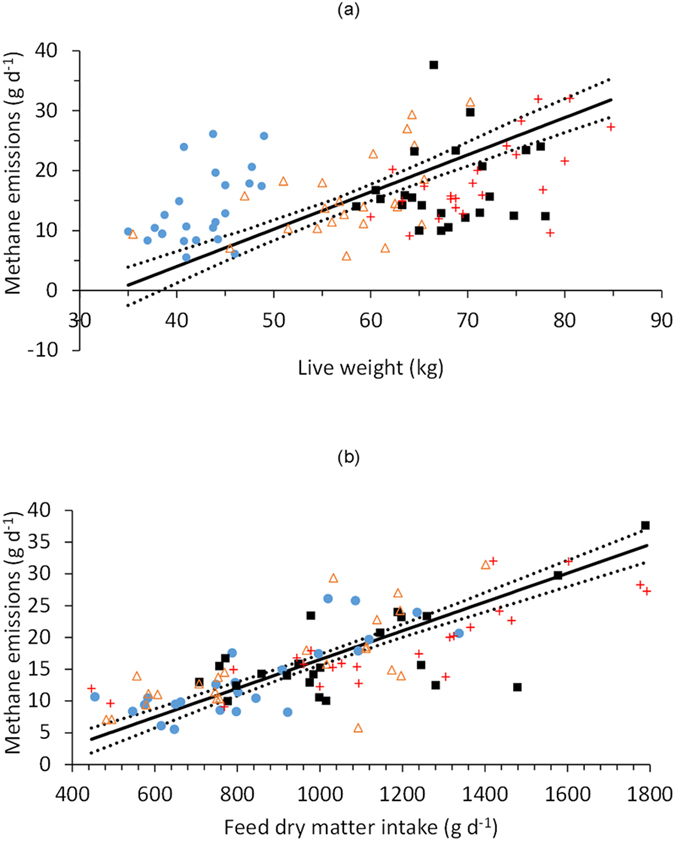
Relationships between (**a**) ewe live weight and methane emissions, and (**b**) ewe feed dry matter intake and methane emissions. The solid line is the linear functional relationship, and the dotted lines are the 95% confidence intervals. Four breeds were used: Welsh Mountain (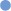), Scottish Blackface (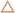), Welsh Mule (■) and Texel (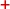).

**Figure 2:**
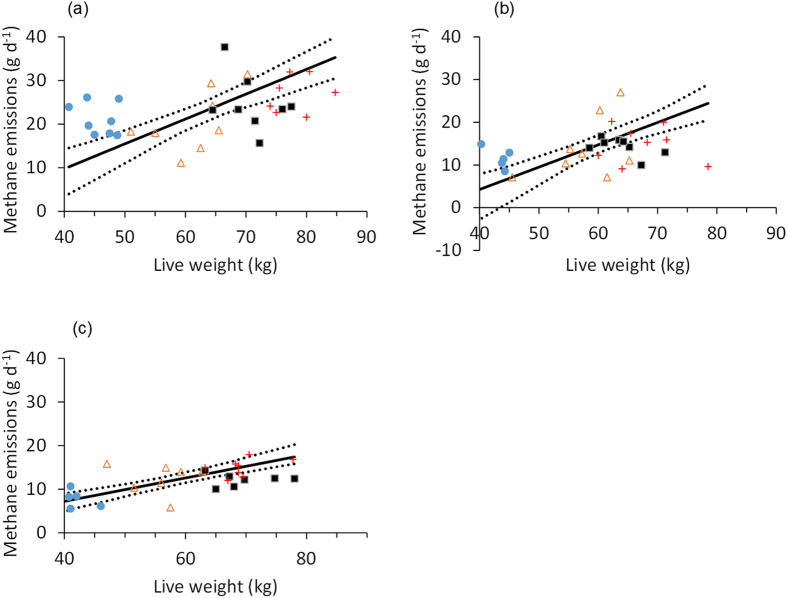
Relationships between ewe live weight and methane emissions when offered (**a**) ryegrass, (**b**) permanent pasture, and (**c**) Molinia. The solid lines are the linear functional relationships, and the dotted lines are the 95% confidence intervals. Four breeds were used: Welsh Mountain (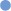), Scottish Blackface (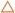), Welsh Mule (■) and Texel (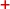).

The overall relationship between LW and *ad libitum* DMI was positive but relatively poor (*r* = 0.52). There was no significant difference in the slopes of this relationship between the different sheep breeds, although there were significant differences in the elevation of the slopes (*P* < 0.001) and the locations of the groups (*P* < 0.001) within the complete dataset. The linear functional relationships between LW and CH_4_ emissions had positive slopes for all sheep breeds (Table 4), but were poorly correlated, particularly for the Welsh Mule ewes (Fig. 1a). There were also poor, but significant, positive correlations between measurements of LW and CH_4_ emissions on each of the three pasture types (Fig. 2), with stronger correlations between DMI and CH_4_ emissions (Table 5), particularly for the ryegrass and permanent pasture diets. The overall linear functional relationship between DMI and CH_4_ emissions was moderate (constant = −6.1 [s.e. = 1.52], slope = 0.022 [s.e. = 0.0015]; *r* = 0.77, *P* < 0.001; Fig. 1b), and there was no significant difference between breed groups in the slopes of the relationships or in the elevation of the regression lines, but there was a significant (*P* < 0.01) difference in the location of the groups.

### Effect of LW on CH_4_ emissions when fed to maintenance requirements

The follow-up experiment investigated the effect of LW on CH_4_ emissions when ewes of the same breeds were fed at energy maintenance. In order to standardise intake levels, and remove between-animal variation in CH_4_ emissions resulting to differences in *ad libitum* DMI, the ewes were offered dried grass pellets at feeding levels formulated to meet predicted maintenance requirements^17^. The mean chemical composition of the grass pellets fed is presented in Table 1. Mean LW ranged from 41 kg for the Welsh Mountain ewes to 70 kg for the Texel ewes. When expressed on a MLW basis the DMI for all breeds was 35 g d^−1^ (s.e.d. = 0.2 g d^−1^; ns). Apparent DM digestibility was similar for all breeds (0.711 g g^-1^ DM; s.e.d. = 0.0207 g g^-1^ DM; ns), and there was no difference in DDMI on a MLW basis (25 g d^−1^; s.e.d. = 0.7 g d^−1^; ns).

There was a significant breed type effect on CH_4_ emitted per day (12.6, 18.5, 17.3 and 19.8 g d^−1^ for Welsh Mountain, Scottish Blackface, Welsh Mule and Texel respectively; s.e.d. 1.09 g d^−1^; *P* < 0.001), with those from the Welsh Mountain ewes being significantly (*P* < 0.05) lower than those from the other breeds. A significant breed type effect was also found when emissions where expressed on a DMI basis (22.1, 25.3, 21.1, 23.4 g kg^−1^ DM for Welsh Mountain, Scottish Blackface, Welsh Mule and Texel respectively; s.e.d. 1.32 g kg^−1^ DM; *P* < 0.05), with emissions from the Scottish Blackface being significantly higher than from the Welsh Mule. The calculated values for Y_m_ followed a similar pattern (7.0, 8.1, 6.7 and 7.4% for Welsh Mountain, Scottish Blackface, Welsh Mule and Texel respectively; s.e.d. 0.42%; *P* < 0.05).

**Figure 3:**
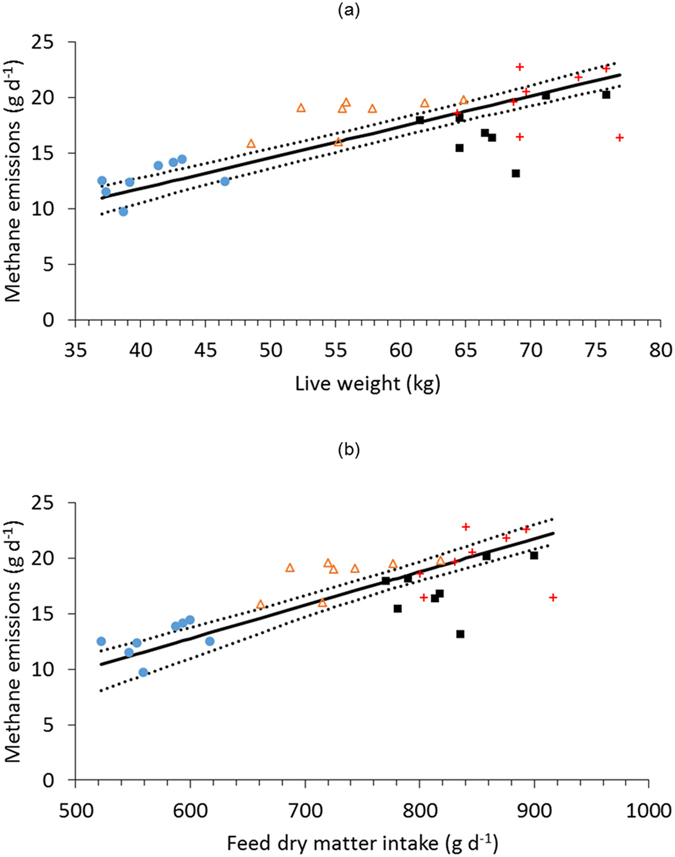
Relationships between (**a**) ewe live weight and methane emissions, and (**b**) ewe feed dry matter intake and methane emissions, of ewes fed dried grass pellets to predicted energy maintenance requirements. The solid line is the linear functional relationship, and the dotted lines are the 95% confidence intervals. Four breeds were used: Welsh Mountain (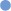), Scottish Blackface (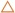), Welsh Mule (■) and Texel (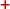).

Restricting intakes of grass pellets to maintenance requirements produced a reasonable overall relationship between LW and CH_4_ (Table 4; Fig. 3a), because individual allocations of feed offered (and consumed) were based on the animals’ LW, i.e. DMI was controlled by LW. Within each sheep breed, the correlations between LW and CH_4_ were generally very poor. There was no significant effect of breed on the slope of the functional relationship, but there were significant differences in the elevation of the regression lines (*P* < 0.001) and in the location of the groups (P < 0.001). The overall linear functional relationship between grass pellet DMI and CH_4_ emissions was moderate (constant = −5.2 [s.e. = 2.11], slope = 0.03 [s.e. = 0.003]; *r* = 0.77, *P* < 0.001; Fig. 3b), and there was no significant difference between breed groups in the slopes of the relationship, although there were significant effects of elevation (*P* < 0.001) and location (*P* < 0.001).

## Discussion

The goal of the current study was to establish the extent to which body size of different UK sheep breeds and associated parameters such as feed intake influence CH_4_ emissions from sheep consuming well defined grass-based diets of differing nutrient concentrations. A zero-grazing approach was chosen to minimise potential confounding of differences in enteric CH_4_ production with differences in grazing behaviour. Ruminant diets have a clear effect on CH_4_ production^18^, but it was not possible to compare between pasture types in this study because the three diets were offered to the sheep at different times, and thus pasture effects would be potentially confounded with differences in ambient conditions and the metabolic status of the ewes. The experimental design with forages fed in separate experimental runs was chosen to allow data collection over as short a time period as possible for each forage in order to minimise confounding with changes in sward chemical composition.

When the ewes were offered the permanent pasture and Molinia voluntary DMI increased with LW, as expected given the greater nutritional demands in absolute terms of larger animals, with intakes of the different breed types then similar when they were expressed on a MLW basis. When offered ryegrass the intake of the Scottish Blackfaces ewes was lower than that of the Welsh Mountains and Texels even on a MBW basis, but a higher apparent digestibility compensated for this. Thus, the DDMI per unit MBW was similar for all breed types regardless of the nutritional characteristics of the particular forage offered. Methane emissions from sheep are known to increase with increasing DM digestibility^19^, because as more of the feed is fermented, more CH_4_ is produced. This explains the higher CH_4_ yields (g CH_4_ kg^−1^ DMI) from the more digestible pastures in the present study, which fall within the range of previously observed values of forages fed to sheep^20^. Changes in the diets of ruminants to improve digestibility are often confounded with changes in the diet components, such that, for example, increasing diet digestibility by increasing the proportion of cereals, can reduce CH_4_ emissions. However, this is because the starch present in cereals is known to reduce the production of CH_4,_ compared to the fibres present in large proportions in most forages, through changes in rumen fermentation patterns^21^,^22^. In the present study, the diets were all cut from grass swards and differences in DM digestibility were limited to differences in basic grass composition. Sheep breed type only affected the amount of CH_4_ emitted per head per day when the ewes were offered the Molinia, the least digestible of the three forages fed, suggesting a limited effect of sheep genetics on CH_4_ emissions^23^. The lower CH_4_ emissions from the Welsh Mountain ewes relative to the Texel ewes when offered this forage mirrors breed differences in DMI.

As *ad libitum* DMI across the range of fresh forage diets increased, the rate of increase in CH_4_ output was similar for all four different breeds of sheep. Even though feed intake scales with LW, because energy and protein requirements for maintenance of body processes increase as body size increases^17^, the relationship between LW and CH_4_ emissions was positive but only moderately correlated. This agrees with previous work in which castrated male sheep were offered dry diets (chaffed oaten hay and cracked lupins) at *ad libitum* rates^24^, compared to studies in which the correlation between feed intake and daily CH_4_ production was much higher at restricted levels of intake^11^,^12^. In this study heavier ewes consumed more feed than lighter ewes, and the response in feed intake to LW was not different between breeds. Thus, although CH_4_ emissions are generally linked to body size by feed intake–with larger animals eating more than smaller ones-the relationship between CH_4_ production and LW in this study was weak when the ewes were fed pasture *ad libitum*, and therefore is unlikely to be of value for improving UK national GHG inventory reporting. Although the nutrient requirements of ewes within commercial flocks change throughout the year due to factors such as pregnancy and lactation, the timing of the current study was representative of when most mature UK ewes would be barren and dry.

When the sheep were fed the common diet of grass pellets according to predicted maintenance energy requirements (based on LW), there was stronger correlation to CH_4_ emissions, but there was still a large amount of individual animal variation in the amount of CH_4_ produced for a given amount of feed consumed. Such variation between individual sheep not attributable to feed composition has been observed previously^16^,^25^,^26^, and contributes significantly to the uncertainty in the estimates of CH_4_ emissions for national inventory reporting^27^. However, such variability among individuals, which likely has a genetic basis^28^, also indicates the potential for breeding livestock with reduced methane emissions^29^.

When CH_4_ emissions from the pasture-fed animals were multiplied up to give annual values, as used in the Tier 1 IPCC inventory approach^30^, the EFs recorded when the ewes were offered ryegrass were broadly in keeping with the value for sheep quoted by the IPCC, whereas those on the permanent pasture and Molinia were substantially lower. A similar pattern was found for the calculated values for Y_m_. The data that IPCC Tier 1 default EFs are derived from are limited^31^, and for example the UK data^32^ that contributed to it was from a small number of sheep fed a restricted diet of pellets made mostly of straw, sunflower-seed meal and barley, i.e. very different to *ad libitum* fresh pasture. It is for this reason that country-specific data are required for Tier 2 and 3 methods of inventory reporting.

In summary, regardless of pasture type ewe LW was a relatively poor indicator of DMI and therefore a poor predictor of CH_4_ emissions from sheep of different breed sizes. There was a stronger relationship between DMI and daily CH_4_ emissions. Live weight therefore currently has limited value in the prediction of CH_4_ emissions from mature ewes, and is unlikely to be of benefit in the development of Tier 2 methodologies for agricultural greenhouse gas inventory reporting. There was a large amount of between-animal variability, although this did not differ between breeds, and if a greater understanding of the factors underlying this variability was obtained, LW may become a more useful factor for predicting CH_4_ emissions for inventory calculations.

## Methods

### Animals used

The work described was conducted in accordance with the requirements of the UK Animals (Scientific Procedures) Act 1986 and with the approval of the Aberystwyth University Animal Welfare and Ethical Review Board. Measurements were made on mature barren ewes of four different breed types: Welsh Mountain, Scottish Blackface, Welsh Mule (Welsh Mountain × Border Leicester) and Texel (n = 8 per breed). The Welsh Mountain and Scottish Blackface are hardy hill breeds commonly used in extensive production systems on marginal grasslands. Cross-bred ewes such as the Welsh Mule are larger and more prolific, and are generally used in more intensive production systems based on improved pasture. The Texel breed is a large meat breed valued for its carcass characteristics. Animals were selected from their respective flocks on the basis of LW and uniformity of body condition score^33^. All animals were drenched with an anthelmintic prior to the start of each experiment.

### Experimental treatments and measurements

The experiment comprised two parts: the first part investigated CH_4_ emissions from the sheep feed on freshly cut forages, while the second part investigated CH_4_ emissions from sheep fed dried grass pellets at quantities designed to meet their maintenance energy requirements. The initial study consisted of three separate experimental periods during which the animals were housed and zero-grazed on herbage cut from contrasting sward types: (1) a recent re-seed of monoculture perennial ryegrass (*Lolium perenne*), (2) a permanent pasture with *Holcus lanatus* (38%), *Agrostis* spp. (27%), *Lolium perenne* (15%) and *Festuca* spp. (15%) as the main components, and (3) a *Molinia caerulea*-dominated native grassland (Molinia). The procedure was similar for each experimental period; ryegrass was fed in August to September 2011, the permanent pasture was fed in October to November 2011, and the Molinia was fed in August to September 2012. In each experimental period, following an adaptation period of at least three weeks during which the ewes received their experimental diets both in group pens and then in individual pens (for three days), they were individually housed in one of four calibrated CH_4_ chambers^34^ and data were collected for three consecutive days for each individual animal. Throughout the experiment the ewes were fed on an *ad libitum* basis, with two equal portions offered at 0900 and 1600. Stored forage was kept refrigerated at approximately 4 °C. Fresh water was available continuously. The same group of 32 ewes was used for all three experiments, with the exception of a Scottish Blackface and a Texel ewe being replaced before the permanent pasture experiment, and again before the Molinia experiment. Within a given breed each animal was assigned to one of the 8 chamber runs per forage at random, and during each run the ewes were randomly assigned to individual CH_4_ chambers.

During the follow-up study measurements were made when the same group of four breeds of ewes were fed to maintenance requirements based on LW^17^. Initially the animals were group-penned according to type and offered the grass pellets in combination with grass silage, with the feeding rates for the grass pellets reflecting live weights. Following an initial acclimatisation period of at least two weeks, one ewe of each breed was selected at random and the four animals housed together in a group pen. At this time the silage was withdrawn and the ewes were fed grass pellets alone. After three days the ewes were transferred to individual pens and offered the grass pellets at quantities calculated to meet their individual maintenance requirements based on LW^17^. Following a further three-day acclimatisation period the animals were transferred to the CH_4_ chambers where they were fed their individual rations as two equal portions offered at 0900 and 1600.

The LW of the sheep was recorded prior to them entering the CH_4_ chambers and again as they were removed. The weights of feed offered and feed refused were recorded on a daily basis. Representative sub-samples of the material offered each day were oven dried at 80 °C to constant weight in order to determine DM content. A further sub-sample of the feed offered was collected at each feeding and bulked for each three-day chamber run prior to subsequent analysis to determine chemical composition. Ash was measured by igniting samples in a muffle furnace at 550 °C for 16 h and gross energy was determined by adiabatic bomb calorimetry. Total nitrogen (TN) concentrations were determined using a Leco FP 428 nitrogen analyser (Leco Corporation, St. Joseph, MI, USA). Water-soluble carbohydrate concentrations were measured by an automated anthrone technique^35^. Neutral deter gent fibre and acid detergent fibre were determined using the method of Van Soest *et al*.^36^, adapted for the Gerhardt Fibrecap detergent system (FOSS UK Ltd, Warrington, UK). Digestibility of organic matter in the DM (DOMD) was determined according to the two-stage method of Tilley and Terry (1963), adapted for the ANKOM DAISYII 220 incubator system (ANKOM Technology Corporation, Fairport, NY, USA).

All faeces were collected twice daily (immediately prior to morning and afternoon feeding). The faeces were weighed fresh, and subsamples taken to determine DM. Faecal DM output data, together with feed DM intake data, were used to calculate apparent whole-tract DM digestibilities.

Methane production was determined by comparing the CH_4_ concentrations in air entering and leaving the chambers at a known rate of airflow. An eight port single channel CH_4_ gas analyser (MGA3000; ADC Gas Analysis Ltd, Hoddesdon, UK) was used to determine CH_4_ concentrations in ambient air and in the exhaust gas leaving each chamber on a rotational basis. The analyser was calibrated using gases of known methane concentrations. Ambient gas was sampled from points between each pair of chambers. For the ryegrass zero-grazing experiment the airflow of each chamber’s exhaust pipe was recorded twice daily during the CH_4_ measurement period using an air velocity meter (TA440; TSI Instruments Ltd, High Wycombe, UK). Ten replicate airflow measurements for each chamber’s exhaust pipe were recorded twice daily. For all subsequent experiments air flow data was measured continuously using mini-vane anemometers (MiniVane6, Schiltknecht Messtechnik, Switzerland) attached to a data logger (MSR145, MSR Electronics GmbH, Switzerland).

### Calculations and data analysis

One Texel ewe and one Welsh Mule ewe did not complete the measurements on one pasture type each, and their data were excluded from the study. Methane emissions data were calculated assuming standard temperature and pressure of 0 °C and 101.325 kPa. Statistical data analysis was carried out using Genstat 16^th^ Edition (VSN International Ltd, Hemel Hempstead, UK). Metabolic LW was calculated as mean LW^0.75^. One-way analysis of variance was used to investigate breed differences for each forage type. Pearson’s correlation coefficients were calculated to investigate the correlations between daily CH_4_ emissions and DMI, LW and metabolic LW, and the correlation between individual pen and chamber feed intake values for each feed type. Linear functional relationship (type 2 regression) using the bisector model was used to regress daily CH_4_ emissions on LW and on DMI, with and without the use of sheep breed and pasture type as grouping factors.

## Additional Information

**How to cite this article**: Moorby, J.M. *et al*. Can live weight be used as a proxy for enteric methane emissions from pasture-fed sheep?. *Sci. Rep*. **5**, 17915; doi: 10.1038/srep17915 (2015).

## Figures and Tables

**Table 1:** Mean chemical composition of the feeds offered (n = 8 per feed).

	Ryegrass	Permanent pasture	Molinia	Grass pellets
Dry matter, g kg^−1^	189	116	351	898
Organic matter	915	894	974	937
Crude protein	110	317	128	154
Water soluble carbohydrates	252	52	39	159
Neutral detergent fibre	512	507	745	536
Acid detergent fibre	250	245	375	263
*In vitro* DOMD^a^	734	685	489	676
Gross energy, MJ kg^−1^ DM	18.5	19.5	20.1	18.8

Values are shown in g kg^−1^ DM unless otherwise indicated.

^a^Digestibility of the organic matter, expressed as proportion of DM.

**Table 2:** Voluntary intake and apparent DM digestibility relative to live weight for different breed types of sheep when zero-grazed on pastures of high (ryegrass), medium (permanent pasture) and low (Molinia) nutritional value.

Sward type	Breed type					
	Welsh Mountain	Scottish Blackface	Welsh Mule	Texel	s.e.d.	*F* Prob.
*Ryegrass*						
LW (kg)	45.8^a^	61.5^b^	70.9^c^	77.6^d^	2.29	<0.001
DMI (kg d^−1^)	1.08^a^	1.02^a^	1.30^ab^	1.55^b^	0.102	<0.001
DMI (g kg^−1^ MLW per day)	62^b^	47^a^	53^ab^	60^b^	4.7	<0.05
DMD (g g^-1^)	0.816^a^	0.857^b^	0.806^a^	0.812^a^	0.0137	<0.01
DDMI (g kg^−1^ MLW per day)	51	40	43	48	3.9	ns
*Permanent pasture*						
LW (kg)	41.3^a^	57.9^b^	63.9^bc^	67.6^c^	2.65	<0.001
DMI (kg d^−1^)	0.76	0.77	0.86	1.03	0.109	ns
DMI (g kg^−1^ MLW per day)	45	35	37	44	5.0	ns
DMD (g g^-1^)	0.794^bc^	0.803^c^	0.763^ab^	0.755^a^	0.0134	<0.01
DDMI (g kg^−1^ MLW per day)	37	29	29	33	3.7	ns
*Molinia*						
LW (kg)	40^a^	53^b^	69^c^	69^c^	2.4	<0.001
DMI (kg d^−1^)	0.66^a^	0.89^ab^	1.03^b^	0.95^b^	0.084	<0.01
DMI (g kg^−1^ MLW per day)	41	45	43	40	3.8	ns
DMD (g g^-1^)	0.577	0.571	0.510	0.504	0.0342	ns
DDMI (g kg^−1^ MLW per day)	24	26	23	21	2.6	ns

LW = live weight; DMI = dry matter intake; MLW = metabolic LW; DMD = dry matter digestibility; and DDMI = digestible DMI. Values in rows with different letter superscripts differed significantly (*P* < 0.05).

**Table 3:** Mean methane (CH_4_) yields by different breed types of sheep when zero-grazed on pastures of relatively high (ryegrass), medium (permanent pasture) and low (Molinia) nutritional value.

Sward type	Breed type					
	Welsh Mountain	Scottish Blackface	Welsh Mule	Texel	s.e.d.	*F* Prob.
*Ryegrass*						
^ ^CH_4_ emitted (g d^−1^)	21.1	20.7	24.7	26.4	3.03	ns
^ ^CH_4_ emitted (g kg^−1^ DMI)	19.7	20.1	19.1	17.0	1.30	ns
^ ^EF (kg/yr)	7.7	7.6	9.0	9.7	1.11	ns
^ ^Ym (%)	5.9	6.0	5.7	5.1	0.39	ns
*Permanent pasture*						
CH_4_ emitted (g d^−1^)	10.8	14.0	14.3	15.0	2.09	ns
^ ^CH_4_ emitted (g kg^−1^ DMI)	14.4^a^	17.5^b^	16.9^ab^	14.8^ab^	1.00	<0.05
^ ^EF (kg/yr)	4.0	5.1	5.2	5.5	0.76	ns
^ ^Ym (%)	4.1^a^	5.0^b^	4.8 ^ab^	4.2^ab^	0.28	<0.05
*Molinia*						
^ ^CH_4_ emitted (g d^−1^)	8.9^a^	12.0^ab^	12.0^ab^	14.9^b^	1.21	<0.01
CH_4_ emitted (g kg^−1^ DMI)	14.1	14.5	12.6	16.8	1.86	ns
^ ^EF (kg/yr)	3.2^a^	4.4^ab^	4.4^ab^	5.4^b^	0.44	<0.01
^ ^Ym (%)	3.9	4.0	3.5	4.7	0.52	ns

DMI = dry matter intake; EF = emission factor; and Ym = percentage of feed gross energy intake excreted as methane energy. Values in rows with different letter superscripts differed significantly (*P* < 0.05).

**Table 4:** Regression coefficients (with standard errors) for the linear functional relationships between live weight and methane emissions in four breeds of mature ewes (n = 8 per breed) offered a range of fresh forage diets *ad libitum*, or when offered dried grass pellets offered at restricted intakes formulated to meet individual animal energy requirements.

Group	Constant (g CH_4_ d^−1^)	Slope (kg^−1^)	R^2^
*Fresh forages*			
Sheep breed			
^ ^Welsh Mountain	−48.2 (8.13)	1.45 (0.194)	0.25
^ ^Scottish Blackface	−38.1 (12.90)	0.93 (0.215)	0.28
^ ^Welsh Mule	−55.5 (61.14)	1.07 (0.901)	0.02
^ ^Texel	−53.9 (14.36)	1.02 (0.203)	0.38
All data (no grouping)	−20.8 (3.20)	0.62 (0.053)	0.19
*Dried grass pellets*			
Sheep breed			
^ ^Welsh Mountain	−9.6 (12.60)	0.55 (0.313)	0.25
^ ^Scottish Blackface	−0.4 (12.58)	0.34 (0.224)	0.43
^ ^Welsh Mule	−25.8 (37.06)	0.63 (0.559)	0.16
^ ^Texel	−44.4 (55.01)	0.91 (0.778)	0.01
All data (no grouping)	0.70 (1.44)	0.28 (0.023)	0.56

**Table 5:** Pearson correlations (*r*) and probabilities for associations between live weight (LW), metabolic LW (MLW; LW^0.75^), dry matter intake (DMI) and methane emitted for ewes zero-grazed on contrasting pasture types.

Pasture type fed		*r*			*F* probability	
Perennial ryegrass		CH_4_	DMI		CH_4_	DMI
	DMI	0.74	–	DMI	<0.001	–
	LW	0.39	0.55	LW	0.028	0.002
	MLW	0.39	0.54	MLW	0.030	0.002
Permanent pasture		CH_4_	DMI		CH_4_	DMI
	DMI	0.81	–	DMI	<0.001	–
	LW	0.38	0.29	LW	0.030	0.104
	MLW	0.39	0.30	MLW	0.028	0.100
Molinia		CH_4_	DMI		CH_4_	DMI
	DMI	0.45	–	DMI	0.009	–
	LW	0.50	0.49	LW	0.003	0.004
	MLW	0.50	0.49	MLW	0.004	0.004
